# Intestinal Microbiota and Liver Diseases: Insights into Therapeutic Use of Traditional Chinese Medicine

**DOI:** 10.1155/2021/6682581

**Published:** 2021-04-22

**Authors:** Tingshuai Wang, Shaodong Huang, Cong Wu, Na Wang, Rongzhen Zhang, Minggang Wang, Dewen Mao

**Affiliations:** ^1^Department of Hepatology, The First Affiliated Hospital of Guangxi University of Chinese Medicine, Nanning 530023, Guangxi, China; ^2^School of Chinese Medicine, Hunan University of Traditional Chinese Medicine, Changsha 410208, Hunan, China; ^3^Department of Gastroenterology, Guangxi Orthopedics and Traumatology Hospital, Nanning 530023, Guangxi, China; ^4^Department of Scientific Research, The First Affiliated Hospital of Guangxi University of Chinese Medicine, Nanning 530023, Guangxi, China

## Abstract

Liver disease is a leading cause of global morbidity and mortality, for which inflammation, alcohol use, lipid metabolic disorders, disturbance to bile acid metabolism, and endotoxins are common risk factors. Traditional Chinese Medicine (TCM) with its “holistic approach” is widely used throughout the world as a complementary, alternative therapy, due to its clinical efficacy and reduced side effects compared with conventional medicines. However, due to a lack of reliable scientific evidence, the role of TCM in the prevention and treatment of liver disease remains unclear. Over recent years, with the rapid development of high-throughput sequencing, 16S rRNA detection, and bioinformatics methodology, it has been gradually recognized that the regulation of intestinal microbiota by TCM can play a substantial role in the treatment of liver disease. To better understand how TCM regulates the intestinal microbiota and suppresses liver disease, we have reviewed and analyzed the results of existing studies and summarized the relationship and risk factors between intestinal microbiota and liver disease. The present review summarizes the related mechanisms by which TCM affects the composition and metabolites of the intestinal microbiome.

## 1. Introduction

Diseases of the liver, including viral hepatitis, liver cirrhosis, nonalcoholic fatty liver disease (NAFLD), liver failure, and liver cancer, affect millions of individuals in China, now representing one of the leading causes of morbidity and mortality [1]. While viruses, inflammation, lipid metabolism, immune response, and bile acid are common pathological mechanisms and risk factors affecting the development of liver disease, none of these factors can fully explain its occurrence and progression.

In recent years, the influence of intestinal microbiota on liver disease has become increasingly apparent [2, 3]. There are approximately 10^14^ microorganisms in the human gastrointestinal (GI) tract, which play an important role in maintaining human health [4]. Changes in the abundance, composition, and fundamental function of the gut microbiome can induce or exacerbate liver disease [5–9]. Studies of the influence of GI flora on the pathogenesis of liver disease are critical in this respect, and it may become a novel therapeutic target for the prevention and treatment of liver disease.

Traditional Chinese Medicine (TCM) is an important component of China's ancient and splendid culture and has been in popular use for thousands of years [10, 11]. As oral administration is among the most common methods of administration of TCM, drugs pass along the intestinal tract and establish a relationship with the intestinal flora [12]. Increasing evidence has shown that monomers or compounds of TCMs can regulate the composition of intestinal flora and its metabolites via the “liver-gut” axis, thereby playing an important role in immune regulation, reversing liver fibrosis, promoting liver regeneration, and reducing inflammatory injury through other modalities, delaying or even reversing the progression of liver disease [13–15]. Detailed research focusing on the relationship between TCM and liver disease is required to elucidate the therapeutic mechanism(s) of TCMs.

In the present review, we first summarize the interactions between intestinal flora and liver disease. We will then review the relationship between intestinal flora and risk factors for liver disease, including inflammation, alcohol use, lipid metabolism, and bile acid metabolism. Lastly, we will summarize the regulatory effect of TCM on intestinal microbial composition and metabolites and illustrate the related mechanisms by which the influence of TCMs on intestinal microbiota prevents and treats liver disease.

## 2. Interaction between Intestinal Microbiota and Liver Disease

The liver-gut axis is an important link between gut microbiota and liver disease. Since 70% of the liver's blood supply comes from the gut via the portal vein, it is constantly at risk of exposure to gut bacterial components such as DNA, metabolites, toxins, lipopolysaccharide (LPS), and peptidoglycans [16, 17]. Intestinal flora is also an important element of the intestinal barrier system, playing a critical role in delaying the release of toxic substances into the bloodstream and maintaining intestinal homeostasis. In addition, intestinal flora participates in the bioconversion of bile acid and regulates hepatointestinal circulation. Trimethylamine (TMA), a metabolite derived from intestinal flora, can be converted into trimethylamine oxide (TMAO) in the liver, which not only damages hepatocytes, but also results in choline deficiency and hepatic steatosis, increasing the risk of liver cancer. Intestinal microbiota may, therefore, be critical in maintaining liver-gut axis homeostasis that limits the pathogenesis of liver disease.

### 2.1. Viral Hepatitis

Studies have found that changes in diversity and structure in the gut microbiome play important roles in inducing and promoting the development of viral hepatitis [18, 19]. Two recent studies have revealed the relationship of HBV, HCV with the intestinal microbiota, in addition to its link with CHB and CHC [20, 21]. It has been found that, in HBV-induced chronic liver disease (HBVCLD) and HCV-induced chronic liver disease (HCVCLD), bacterial flora diversity had declined, with a notable decrease in *Bifidobacterium* and *Clostridiaceae*, and increased *Enterobacteriaceae* family bacteria (such as *Streptococcus* and *Lactobacillus*). *Bifidobacteria* are important probiotics that reduce endotoxin levels in the plasma and intestine, maintain intestinal microbial homeostasis, produce antibacterial factors, improve intestinal barrier function, and regulate systemic immunity [22, 23]. *Clostridiaceae* are an additional group of probiotics that represents the main commensal and butyrate-producing group of bacteria. *Enterobacteriaceae* are nondominant flora, the excessive growth of which may release large quantities of endotoxin into the intestinal tract, inhibiting protein synthesis in intestinal epithelial cells that leads to intestinal barrier injury, bacterial translocation, and floral imbalance [24]. In addition, changes in the composition of gut microbiota are also associated with the production of bile acid, the reduction of which can lead to the excessive growth of pathogenic and proinflammatory bacterial species such as *Enterobacteriaceae* and *Porphyromonadaceae* [25].

Hepatic cells are destroyed by HBV or HCV infection, negatively impacting the homeostasis of the liver-gut axis and leading to other dangerous consequences (such as inflammation or immune injury). Thus, determining the composition of the microbiome provides a new tool for establishing the relationship between diseases caused by viral infection and inflammation, immunization, and bile acid metabolism.

### 2.2. Nonalcoholic Fatty Liver Disease (NAFLD)

Obesity and a high-fat diet (HFD) are important risk factors for NAFLD and are associated with qualitative and quantitative changes in the intestinal flora. In a prospective, cross-sectional study that compared the gut microbiome in patients with NAFLD with healthy subjects, 20% more *Bacteroidetes* and 24% fewer *Firmicutes* were observed in the NAFLD group. The reduction in *Firmicutes* included 8 genera of SCFA-producing bacteria, including *7α-dehydroxylating Ruminococcaceae* [26]. It has been demonstrated that SCFAs reduce the quantity of stored lipid, thus altering hepatic metabolic processes. SCFA levels (including acetic acid, propionic acid, butyrate, propionate, and butyrate) in NALFD mice were significantly higher following their treatment with Silybin (a hepatoprotective agent), possibly related to changes in the intestinal flora, especially the significant decrease in *Firmicutes* and *Bacteroides* [27]. Lipid metabolism can affect endogenous ethanol (EnEth) fermentation, a process in which certain gut bacteria (*HiAlc K. pneumoniae*) are involved, promoting the development of NAFLD. Chen et al. found that serum aspartate transaminase (AST), alanine transaminase (ALT), triglycerides (TG), and malondialdehyde (MDA) levels increased when the alcohol production capacity of the high alcohol-producing (HiAlc) *Klebsiella* pneumonia strain W14 increased, with lipid accumulation severely damaging liver tissue, as observed in histology [28]. In addition, TMAO affects bile acid metabolism and exacerbates hepatic steatosis. It was observed that TMAO levels in patients with NAFLD positively correlated with BA levels in serum and BA synthesis. This suggests that TMAO leads to increased BA synthesis and regulates BA composition toward FXR antagonistic activity, thereby inhibiting the activation of FXR in the liver, consequently improving lipogenesis [29].

NAFLD is an extremely complex disease that arises from interactions between the gut microbiota, diet, genes, and environment. In addition to directly inducing NAFLD, the gut microbiota can drive the severity of NAFLD and promote nonalcoholic steatohepatitis (NASH), liver fibrosis, liver cirrhosis, and even liver cancer. The prevention and treatment of NAFLD via regulation of the microbiome are a promising area of research for the identification of new therapeutic targets.

### 2.3. Liver Cirrhosis

Intestinal changes frequently occur in cirrhotic patients that can include intestinal bacterial overgrowth, bacterial translocation (BT), disruption to the intestinal barrier, and increased levels of endotoxins [30, 31]. A prospective study found that microbial diversity decreased in cirrhotic patients, and it is even lower in those with decompensated cirrhosis. As the disease progresses, a progressively increased abundance of *Enterococcaceae* and *Enterobacteriaceae* has been observed to be positively correlated with Model for end-stage liver disease (MELD) scores and endotoxin levels [32, 33]. SCFAs are an important source of energy for intestinal epithelial cells and can enhance the intestinal barrier by promoting cell differentiation, production of mucin and antimicrobial peptides, and upregulation of tight junction proteins [34]. SCFAs also have an anti-inflammatory effect by inhibition of NF-*κ*B activity. *Lactobacillus* and *Eubacterium*, the principal SCFA-producing genera, are significantly reduced in cirrhosis patients [35]. Bile acids play a pleiotropic role in the maintenance of intestinal barrier homeostasis. Animal models of bile acid deficiency have allowed the observation of the effects of intestinal bacterial overgrowth, inflammation, increased permeability, and BT on mesenteric lymph nodes (MLN) [36].

Cirrhosis is preceded by fibrosis and eventually develops into liver cancer. Fundamental and clinical research into cirrhosis has also examined the microbiome [37–39], aiming to identify key microorganisms and their metabolites, and examining the effects on BT and intestinal permeability. By elucidating the relationship between the microbiome and liver cirrhosis, the eventual aim is to utilize this knowledge to reverse or inhibit cirrhosis and prevent hepatocellular carcinoma (HCC).

### 2.4. Liver Failure

Qualitative and quantitative changes to the microbiome, including dysbiosis and bacterial overgrowth, are closely linked to liver failure, due to hepatocyte apoptosis, inflammatory reactions, and endotoxemia, which exacerbate liver damage. The imbalance in intestinal microflora accelerates the progression of liver failure. Chen et al. [40] found that ACLF patients had a lower abundance of *Bacteroidaceae*, *Ruminococcaceae*, and *Lachnospiraceae* and a higher abundance of *Pasteurellaceae*, *Streptococcaceae*, and *Enterococcaceae*. Through correlation-network analysis, it was found that TNF-*α* was negatively correlated with *Proteobacteria*, *Burkholderiales*, and *Bacteroidetes* and that the relative abundance of *Oscillibacter spp*., *Butyricimonas spp*., and *B. virosa* was positively associated with levels of IL-1*β*, TNF-*α*, and chemokines such as GM-CSF, CXCL1, MIP-1*α*, CCL5, and MCP-1. In addition to the inflammatory response, gut microbiota also affects the progression of liver failure. One study found that, in ACLF patients, the number of immunomodulatory monocytes and MERTK-expressing macrophages increased, inhibiting the innate immune response to microorganisms [41].

Liver failure is an important clinical challenge as a highly prevalent, life-threatening disease with few current therapeutic options available. Although the pathophysiology of liver failure still requires elucidation, the gut microbiota and associated metabolites, in addition to BT, are thought to be all major contributing factors [42, 43]. Therefore, future research should focus on methods of improving intestinal flora with the overall aim of regulating intestinal homeostasis.

### 2.5. Liver Cancer

Recent studies indicate that changes in the composition of the intestinal microbiota and their metabolites also represent a significant risk factor for HCC [44, 45]. Intestinal microbiota have multiple roles and can regulate the immune response, while also promoting the development of cancer and the anti-tumor response following immunotherapy and chemotherapy [16, 46]. A large, cross-regional report [45] from China found that fecal microbial diversity decreased in patients with liver cirrhosis compared with healthy subjects, although it was greater in patients with liver cirrhosis compared with those with early HCC. This suggests that altered microbial communities may play an important role in the occurrence and development of HCC. Compared with patients with liver cirrhosis, 13 genera, including *Gemmiger*, *Parabacteroides*, *Paraprevotella*, and *Clostridium XVIII*, were enriched in early HCC patients. In patients with early HCC, *Ruminococcus*, *Oscillibacter*, *Faecalibacterium*, *Clostridium IV*, and *Coprococcus* (which produce butyrate) were lower, while *Klebsiella* and *Haemophilus* (which can produce LPS) were higher. The damage to the intestinal mucosa and barrier function in early HCC is related to a decrease in butyrate-producing bacteria and increased levels of LPS-producing bacteria. Deoxycholic acid (DCA) is a metabolite of intestinal bacteria such as *Clostridium cluster XI* and *XIVa*, which can cause DNA damage, playing a key role in the development of obesity-related HCC [47]. A case-control study reported that higher serum concentrations of TMAO were associated with increased HCC risk. Higher levels of serum choline resulted in a lower risk of PLC [48].

HCC is an end-stage liver disease with high mortality, limited effective treatments, and a low clinical cure rate. Understanding the microbiome may be of great value to research into the mechanism, early diagnosis, and clinical treatment of HCC. Therefore, the study of intestinal flora in HCC is of critical importance and may provide potential target(s) for adjuvant therapy in HCC treatment regimes.

## 3. Link between Gut Microbiota, Liver Disease, and Related Risk Factors

**Figure 1:**
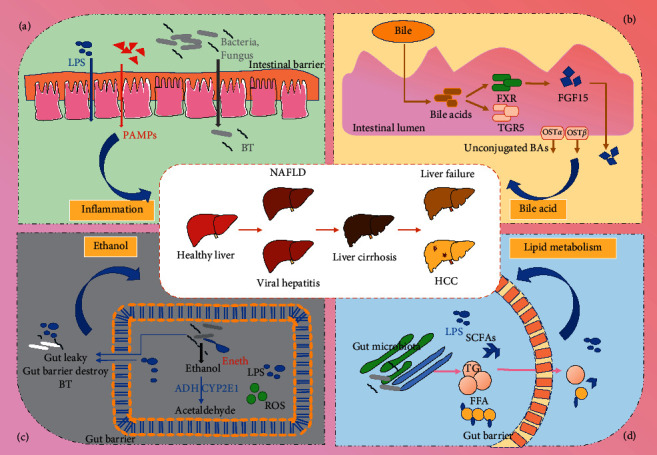
The link between gut microbiota and liver disease risk factors.Inflammation, EnEth, lipid metabolism, bile acid metabolism, and endotoxin levels are the common risk factors for liver diseases induced by intestinal microbiotas and their metabolites. (a) Inflammation: it is one of the most typical features of liver disease and occurs at all stages of disease development. (b) Bile acid metabolism: bile acid is an important part of bile, and intestinal floras regulate its metabolism through the FXR/TGR5 pathway. (c) Ethanol: in addition to exogenous alcohol intake, intestinal flora also can produce a large amount of ethanol (EnEth), affecting intestinal barrier and BT. (d) Lipid metabolism: gut microbiota influence on lipid metabolism may be mediated through metabolites and LPS. NAFLD, nonalcoholic fatty liver disease; HCC, hepatocellular carcinoma; LPS, lipopolysaccharides; PAMPs, pathogen-associated molecular patterns; BT, bacteria translocation; FXR, farnesoid X receptor; EnEth, endogenous ethanol; ROS, oxidative stress, and reactive oxygen species; SCFAs, short-chain fatty acids; TG, triglycerides; FFA, free fatty acids.

The pathophysiology of different liver diseases exhibits some common characteristics. Pathogenic risk factors alter the composition and quantity of gastrointestinal microflora. Factors including inflammation, endogenous ethanol production, fat metabolism, bile acid metabolism, and endotoxin production are influenced by intestinal microbiota, and their metabolites are related to the occurrence and development of liver diseases (Figure 1). Where the expression of proinflammatory factors in the liver is promoted, it causes activation of intestinal inflammation, damage to the intestinal barrier, and promotion of the development of endotoxins and their release into the blood, resulting in further progression of the liver disease (Table 1).

### 3.1. Inflammation

Inflammation is among the most common symptoms of viral, alcoholic, or fatty liver disease. It occurs at all stages and is closely related to fibrosis, cirrhosis, cancer, and failure of the liver [50]. Microbial pathogens or danger signals from the host can trigger inflammation. The hepatitis virus is a common cause of liver inflammation, and endogenous bacteria are responsible for the majority of liver diseases [51].

Local inflammation, systemic inflammation (SI), endotoxemia, and complications such as SBP and HE are all closely linked to the gut microbiota [52]. Infiltration of inflammatory cells is common in chronic liver disease. A murine model of NAFLD indicated that there was an increase in SI combined with increased levels of TNF-*α* and MCP-1 [53]. According to the systemic inflammation hypothesis, chronic inflammation induces the occurrence of ACLF due to the translocation of PAMPs from the intestine and/or DAMP production derived from the damaged liver. Claria et al. [54] found that the severity of SI exhibited positive correlation with ACLF severity and that IL-8, IL-6, and HNA2 levels were directly correlated with the 28- and 90-day mortality of patients. Recent human and animal studies have demonstrated that changes in the relative abundance and composition of the intestinal microbiome affect inflammation and the manifestation of liver disease, and its prognosis. Increased BT is another feature of chronic and end-stage liver disease, which not only contributes to the entry of bacteria and their components, such as LPS, into the portal vein circulation, but also increases the expression of specific receptors such as TLR4 that activates the inflammatory factors TNF, IL-1, and IFN [55].

### 3.2. Ethanol

In the 1850s, scientists discovered that the human body naturally produces small quantities of ethanol, termed EnEth [56]. Ethanol has a direct toxic effect on the liver and can cause hepatocyte degeneration and necrosis. Patients with liver injury were found to have a higher prevalence of intestinal diseases, EnEth production, intestinal permeability, and bacterial translocation [28, 57]. In addition to being a hepatotoxin, there is considerable evidence that ethanol is also a carcinogen [57].

The increased abundance of *Firmicutes* and *Bacteroidetes* was observed in patients with NASH and obesity, which was accompanied by higher serum ethanol levels, indicating that *Escherichia coli* may contribute to elevated blood ethanol levels [58]. Increased microbial ethanol production may lead to a chronic, low-level exposure to the hepatotoxin, exposing the body to the risk of inflammation and hepatitis. A recent study indicated that EnEth produced by HiAlc bacteria is an important factor in the induction of lipid accumulation and mitochondrial damage in hepatocytes [28]. In the absence of a history of liver disease, acute and high levels of alcohol intake can also cause damage to the intestinal mucosa and barrier, increasing the level of bacteria and related components. In addition, alcohol is a recognized carcinogen, the risk of which starts at a dose of 10 g/1 unit/day. Acetaldehyde formation leads to the direct damage to proteins and DNA, while increased ROS production further exacerbates damage to antioxidant defense and DNA repair mechanisms. Changes in ethanol levels can also affect the immune system and induce chronic inflammation, all of which are related to the mechanisms of alcohol-mediated HCC [57].

### 3.3. Lipid Metabolism

The liver plays an important role in the synthesis, decomposition, metabolism, and storage of lipids and lipoproteins. In general, the levels of lipids and lipoproteins decrease with the increasing severity of liver disease [59]. However, specific analyses should be performed depending on disease etiology. Differences in the abundance, structure, and quantity of the gut microbiome influence the regulation of lipids and related metabolites.

Just et al. found that the decreased relative abundance of *Lachnospiraceae* was associated with reduced cholesterol biosynthesis and elevated cholesterol levels in the liver [60]. In contrast, An et al. found that *Lactobacillus* and *Bifidobacterium* reduce cholesterol, triglycerides, and LDL levels in the blood circulation, in addition to increasing HDL levels [61]. Changes in the intestinal flora as NAFLD progresses can interfere with hepatic lipid metabolism, the promotion of lipid accumulation, and lipotoxicity [62]. Levels of free fatty acids (FFAs) and bacterial metabolites such as LPS can activate TLR4 and induce the transduction of relatively proinflammatory signaling pathways [55]. In liver cirrhosis, it is common for lower levels of cholesterol, triglycerides, and lipoproteins, but the relationship between the severity of cirrhosis and lipid levels varies and is dependent on the different underlying causes of the disease. HDL concentrations in patients with nonalcoholic liver cirrhosis increase in accordance with disease severity, whereas LDL levels decrease with disease progression. In alcoholic liver cirrhosis, only triglyceride levels change as the disease progresses [63].

### 3.4. Bile Acid Metabolism

Intestinal blood accounts for 70% of the whole liver blood supply, and bile acids (BAs) can be recycled back to the liver through the liver and intestines, indicating that BAs link gut microbiota to liver and intestinal metabolism, thereby affecting gastrointestinal motility, intestinal permeability, and carcinogenesis.

The quantity of lactobacillus DNA in fecal samples has been shown to display a positive correlation with the severity of liver steatosis in mice, suggesting that *Lactobacillus* may increase the risk of fatty liver disease by influencing lipid metabolism through their impact on bile acid metabolism [64]. The similarity between the change in intestinal microbes in HFD-fed rats and NASH patients suggests that HFD may be an important cause of the initiation of elevated intestinal bile acid [65]. An additional study compared gut microbiota and BA levels in healthy patients with NASH-non-HCC and NASH-HCC patients, finding that there was a gradual decrease in the abundance of Bacteroidetes and Actinobacteria, with a progressive increase in *Lactobacillus*. A separate study found that intestinal microbiota use bile acids as messengers to control CXCL16-dependent accumulation of liver NKT cells, which play a role in anti-tumor immunity and are active against primary and metastatic liver tumors [66]. Bile acids play an important role in liver regeneration. In the absence of intestinal bile, liver regeneration in rats has been shown to be impeded, with low levels of bile acids impairing liver regeneration and high levels promoting it [67, 68].

## 4. Intervention Therapy Using Traditional Chinese Medicine (TCM)

**Figure 2:**
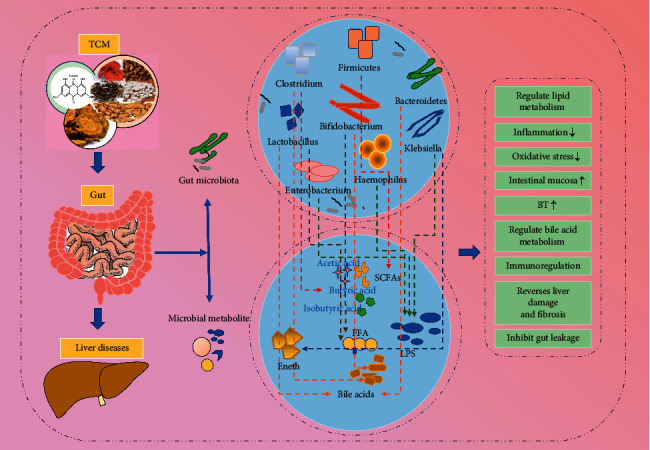
TCM affects liver disease progression by regulating intestinal microbiotas. The composition of intestinal microbiotas and their metabolites can be regulated by TCM. For example, Chinese herbs can increase SCFA levels by promoting abundance of *Clostridium* and *Firmicutes*, elevate bile acid levels by increasing *Lactobacillus* and *Bifidobacterium* abundance, and reduce levels of LPS and inflammatory factors by decreasing populations of *Klebsiella* and *Haemophilus*. The specific mechanisms include reducing inflammatory injury and oxidative stress, regulating body immunity, improving lipid metabolism, protecting intestinal barrier, and reversing hepatic fibrosis. TCM, traditional Chinese medicine; LPS, lipopolysaccharides; BT, bacterial translocation; EnEth, endogenous ethanol; SCFAs, short-chain fatty acids; FFA, free fatty acids.

As a treasure of Chinese culture, TCM is widely accepted in China and East Asia due to its remarkable ability to cure diseases with few side effects. It has become increasingly popular in both Western countries and the rest of the world [69]. A number of studies have demonstrated that individual ingredients or compounds in TCM can inhibit the growth of pathogens, enhance the growth of beneficial bacteria, restore the diversity of intestinal flora, enhance intestinal barrier function, and improve immune function [14, 70–75] (Figure 2, Table 2). In addition, polyphenols, dietary prebiotic fiber, and probiotic components are widely found in TCM and have been found to regulate the gut microbiota.

### 4.1. TCM Regulates the Composition of Intestinal Flora

Oral administration is among the most common methods of receiving TCM, and thus, it will inevitably interact with the intestinal flora [85]. TCM may directly or indirectly regulate the composition of the gut microbiota and maintain its dynamic balance via the promotion, inhibition, or elimination of bacterial growth and new colonization [13].

A number of studies have demonstrated that TCM can affect the progression of liver disease by altering the composition of the intestinal microbiome. Polyphenol-rich loquat fruit extract (LFP) regulates the microbiome by maintaining the ratio of intestinal *Firmicutes*/*Bacteroidetes*, thereby reducing oxidative stress and inflammation, decreasing lipid metabolism disorders, and alleviating high-fructose diet- (HF-) induced NAFLD [76]. Water-insoluble polysaccharides, consumed as prebiotics, isolated from the sclerotium of *Poria cocos*, can increase butyrate production by increasing the abundance of Lachnospiraceae, *Clostridium XIV A* and *IV*, thereby enhancing the integrity of the intestinal mucosa [72]. The abundance of *Clostridium XIV A* and *IV* has been shown to negatively correlate with liver steatosis [86]. In rats with liver cirrhosis induced by carbon tetrachloride (CCl_4_), Artesunate (an extract of *Artemisia annua*) has been shown to increase levels of *Lactobacillus* and *Eubacterium*, thereby reducing the concentration of proinflammatory IL-6 and TNF-*α*, reducing injury to the intestinal mucosa, and decreasing the numbers of translocated bacteria [77]. TCM compounds, such as Qushi Huayu decoction and Erzhi pills, are also capable of modifying the composition of the intestinal flora, which can affect fatty liver disease [73, 87].

### 4.2. TCM Regulates the Metabolism of the Intestinal Flora

The principal gut flora metabolites include SCFAs, secondary bile acid, vitamins, polyphenols, lipids, and indole derivatives, which participate in a variety of biological functions [88, 89]. TCMs can regulate the metabolism of the gut microbiome by modulation of its composition and/or by adjusting the activity of enzymes produced by catalytic metabolites [90]. Because TCM can promote, inhibit, eliminate, or colonize particular bacterial species, it, therefore, has the ability to regulate the metabolites of the intestinal microbiome.

Shenling Baizhu powder was found to enhance the overall abundance of the intestinal microflora in a rat model of NAFLD, and the relative abundance of *Bifidobacteria* and *Anaerobe* also increased, enhancing levels of SCFA [78]. Similarly, Berberine and Stachyose also increase the levels of SCFAs produced by a number of bacteria (*Akkermansia*, *Firmicutes*, and *Bacteroidetes*) in mice fed a HFD [91, 92]. LPS is a glycolipid found on the outer membrane of Gram-negative bacteria, and its increase is known to be an important cause of endotoxemia. Dachengqi decoction, recorded in the Treatise on Febrile and Miscellaneous Disease, is a typical prescription for catharsis. Modern studies have found that it reduces both endotoxin levels and gut inflammation [93]. In a CCl_4_-induced model of chronic liver injury, proteins related to the synthesis (CYP7A1), excretion, and reabsorption of bile acids (such as NTCP, Mrp2, and BESP) were upregulated following the administration of Sanwei Ganjiang powder [79].

### 4.3. Mechanisms of the Regulation of the Gut Microbiome by Traditional Chinese Medicine

Through the theories of TCM as “syndrome differentiation and treatment” and the “concept of wholism,” the relationship between liver diseases and intestinal flora is inseparable. The majority of Chinese herbs exhibit the mechanism (termed “gongxiao” in Chinese) of promoting probiotic action and inhibition of pathogens, with synergy occurring with two or more compatible Chinese medicines [94]. The intestinal flora may, therefore, be a promising novel pharmacological target for the prevention and treatment of liver disease using TCM.

The mechanisms by which Chinese herbs regulate intestinal flora and thus affect the progression of liver disease involve a reduction in inflammatory injury and oxidative stress, which regulate an individual's immunity, reducing ammonia levels in serum, improving lipid metabolism, protecting the intestinal barrier, and reversing hepatic fibrosis, which are closely related to the TLR4 signaling pathway, and the involvement of NF-*κ*B, ROS, NOX4/ROS, and RhoA/ROCK1 [78, 80–84, 91]. Rhubarb extract affects antimicrobial peptide production and the stability of the intestinal environment, via the downregulation of inflammatory and oxidative marker expression, such as TLR4 and NADPH oxidase, which strengthens the intestinal barrier, thereby reducing liver damage caused by acute alcohol intake [80]. The microbial enzyme urease catalyzes the hydrolysis of urea to produce excessive ammonia, usually produced by Gram-negative *Enterobacteriaceae*, a number of anaerobes, and Gram-positive bacteria. Babaodan is able to reduce ammonia levels in the serum and inflammatory cytokines in rats with hepatic encephalopathy, possibly related to the TLR4/MyD88/NK-*κ*B signaling pathway. In animal models treated with a HFD, berberine was able to inhibit *Akkermansia* and increase the number of SCFA-producing bacteria, such as *Lachnospiraceae*, thereby reducing inflammation and improving intestinal epithelial barrier function [91]. Shenling Baizhu powder is able to affect NAFLD progression via an increase in the abundance of beneficial bacteria and a reduction in the level of intestinal endotoxin [78]. In addition to reducing inflammatory injury, immune regulation is another important mechanism by which TCM prevents and treats liver diseases. S-3-1 [81], a homogeneous polysaccharide purified from Sijunzi decoction, has been shown to be immunomodulatory through an adjustment in the abundances of 9 intestinal genera (including *Lactobacillus*, *Pediococcus*, *Sutterella*, *Paraprevotella*, *Bacteroides*, *Streptococcus*, *Clostridium*, *Ruminococcus,* and *Butyricimonas*).

## 5. Conclusions and Perspectives

The discovery that gut flora has a significant metabolic influence in humans has opened a new paradigm in modern medical research. The processes of birth, aging, disease, and death are influenced by the balance of intestinal microbiota, which is related not only to intestinal health, but also to liver, brain, kidney, and organismal homeostasis. Additionally, researchers are now investigating the gut flora as a starting point for the exploration of biomarkers and treatments for many types of disease (such as cardiovascular diseases and liver disease) beyond the gut itself. This exploration is consistent with the Chinese approach of “holistic concept.” Intestinal flora and their metabolites participate in the development of liver diseases, suggesting that the regulation of intestinal flora with TCM can play an important role in the treatment of liver disease. However, using the guiding TCM principle of “syndrome differentiation and treatment” and the overall concept of personalized therapy, it is necessary to systematically identify the differences in intestinal flora and their metabolites for the same TCM type of different liver diseases and different TCM types for the same disease. This will assist in establishing the mechanism by which intestinal microorganisms transform drugs into metabolites. Extensive DNA sequencing studies have expanded our understanding of the ecological and biological characteristics of the gut microbiota. Improved computational techniques, high throughput DNA sequencing, and study of the blood metabolome have deepened our understanding of gut microbiota. However, methodological differences among researchers have led to inconsistent data. Therefore, it is necessary to establish systematic and standardized research methods to study intestinal flora. Chinese herbs treat liver disease via regulation of the composition and metabolism of intestinal flora and have shown good clinical efficacy. Future research should focus on individual treatments and characterization of the relationship between intestinal flora, metabolites, and TCM's curative effect. In addition, due to the lack of active therapeutic effects of TCM on liver disease and an analysis of the specific mechanisms, further research (including basic research and clinical research) is still required.

## Figures and Tables

**Table 1:** Interaction between the intestinal microbiota and liver diseases.

Disease	Subjects	Gut microbiota	Gut microbial metabolites	Potential mechanisms	Ref.
Viral hepatitis	CHC patients	↑*Streptococcus* and *Lactobacillus*; ↓*Clostridiales*	↑pH	Gut dysbiosis, *Viridans streptococci*↑	Inoue et al. [20]
	HBVCLD (CHB, LC) patients	↑Enterobacteriaceae, *Bacteroidetes*/*Firmicutes*, *Proteobacteria*; ↓*Bifidobacteriaceae*, *Clostridiaceae*, *Actinobacteria*	None	Glycan biosynthesis↑, potential bacteria↑, potential beneficial bacteria↓	Zeng et al. [21]

NAFLD	NAFLD patients	↑*Bacteroidetes*, Gram-negative/Gram-positive; ↓*Firmicutes*/*Bacteroidetes*, *Firmicutes*, *Lachnospiraceae*, *Ruminococcaceae*, *Lactobacillaceae*, *Peptostreptococcaceae*	↓SCFAs	SCFAs-producing bacteria↓, beneficial bacteria↓, potentially opportunistic pathogenic lipopolysaccharide-producing bacteria↑	Wang et al. [26]
	HFD-induced NAFLD mice	↑*Lachnoclostridium*, *Lachnospiraceae_UCG-006*, *Mollicutes_RF9*; ↓*Blautia*, *Akkermansia*, *Bacteroides*	↑lithocholic acid, deoxycholic acid; ↓acetate, propionate, butyrate	Changes in secondary metabolites (bile acid and SCFAs)	Li et al. [27]
Liver cirrhosis	In vitro	↑*Hi Alc K. pneumoniae*	↑EnEth	Mitochondrial dysfunction↑, lipid accumulation↓	Chen et al. [28]
	Liver cirrhosis patients	↑ *Enterococcaceae*, *Staphylococcaceae*, *Enterobacteriaceae*; ↓*Clostridiales XIV*, *Ruminococcaceae*, *Lachnospiraceae*, *Veillonellaceae*, *Porphyromonadaceae*,	None	CDR↓, LPS↑, inflammatory response↑	Bajaj et al. [32]
	Liver cirrhosis patients with/without HE patients	↑*Staphylococcaceae*; *Enterococcaceae*, *Porphyromonadaceae*, *Lactobacillaceae*; ↓*Lactobacillus*, *Eubacterium*	↑endotoxin, ammonia; ↓SCFAs	Systemic inflammation↑, ammonia↑, neuronal and astrocytic dysfunction↑	Ahluwalia et al. [35]

Liver failure	ACLF patients	↑*Pasteurellaceae*, *Streptococcaceae*, *Enterococcaceae*; ↓*Bacteroidaceae*, *Ruminococcaceae*, *Lachnospiraceae*	None	Systemic inflammation↑	Chen et al. [40]
	Rat model of acute liver failure	↑*Acetatifactor muris*, *Butyricimonas spp.*, *Oscillibacter spp.*; ↓*Alloprevotella spp.*	None	probiotics↓	Wang et al. [49]

Liver cancer	Liver cancer patients	↑*Klebsiella*, *Haemophilus*; ↓*Alistipes*, *Phascolarctobacterium*, *Ruminococcus*, *Oscillibacter*, *Faecalibacterium*, *Clostridium IV*	↑LPS, ↓butyrate	LPS-producing bacteria↑; butyrate-producing bacteria↓	Ren et al. [45]
	Obesity-associated HCC mice	↑*Clostridium cluster XI*, *XIVa*	↑deoxycholic acid	Gram-positive bacteria↑	Yoshimoto et al. [47]

CHC: chronic hepatitis C; HBVCLD: HBV-induced chronic liver disease; SCFAs: short-chain fatty acids; NAFLD: nonalcoholic fatty liver disease; HFD: high-fat diet; EnEth: endogenous ethanol; BT: bacteria translocation; LPS: lipopolysaccharide.

**Table 2:** Mechanism by which TCM regulates the composition and metabolism of intestinal flora.

Herbs/decoction	Source/component	Model	Microbial target	Microbial metabolite	Related mechanism	Ref.
Water-insoluble polysaccharide	*Poria cocos*	ob/ob mice	↑ *Lachnospiraceae*, *Clostridium*, *Bacteroidetes*, *Alloprevotella*, *Parabacteroides*, *Clostridium IV*, *Ruminococcus*, *Bacteroides*; ↓*Megamonas*, *Proteus*	↑SCFAs, butyrate	Regulate lipid and glucose metabolism	Sun et al. [72]
Qushi huayu decoction	*Artemisiae scopariae herba*, *Polygoni cuspidati rhizome et radix*, *Curcumae longae rhizome*	High-fat diet	↑*Clostridium XIV a*, *Clostridium IV Odoribacter*; ↓*Rikenella*, *Tyzzerella*, *Intestinibacter*, *Romboutsia*, *Lachnospiraceae*	↓LPS	Inhibit LPS gut-leakage, downregulate intestinal MAPK pathway	Leng et al. [73]
Polyphenol-rich loquat fruit extract	Loquat fruit	High-fructose diet	↑*Bacteroidetes*, *Firmicutes*, *Proteobacteria*, *Actinobacteria*; ↓*Firmicutes/Bacteroidetes ratio*	None	Reduce oxidative stress and inflammation, decrease lipid metabolism disorders	Li et al. [76]
Artesunate	*Artemisia annua*	CCl4-induced liver cirrhosis	↑*Lactobacillus*, *Eubacterium*	None	Reduce injury to intestinal mucosa, decrease translocated bacteria	Chen et al. [77]
Shenling Baizhu powder	*Ginseng radix et rhizoma*, *Poria, Atractylodis macrocephalae rhizoma*, *Dioscoreae rhizoma*, *Lablab semen album*, *Nelumbinis semen*, *Glycyrrhizae radix et rhizoma praeparata cum melle*, *Coicis semen*, *Platycodonis radix*, *Amomi fructus*	HFD-induced NAFLD	↑*Bifidobacteria*, *Anaerobe*	↑SCFAs, ↓LPS	Inhibit TLR4/MYD88 pathway	Zhang et al. [78]
Sanwei ganjiang powder	*Zingiberis rhizoma*, *Alpinia katsumadai*, *Myristica fragrans houtt*	CCl4-induced chronic liver failure	↑*Firmicutes*, *Lactobacillus*; ↓*Firmicutes/Bacteroidetes ratio*, *Bacteroidetes*, *Actinobacteria*, *Coprococcus*, *Ruminococcus*, *Sutterella*	↑CYP7A1, NTCP, Mrp2, BESP	Regulate bile acid metabolism, increased the expression of Nrf2, decrease inflammatory response	Li et al. [79]
Rhubarb extract	*Rheum palmatum*	Mouse model of binge drinking	↑*Parabacteroides goldsteinii*; ↓*Lachnospiraceae*, *Prevotellaceae*, *Akkermansia*,	↓LPS	Improve gut barrier function, relieve oxidative stress and inflammation, inhibit TLR4 and NADPH oxidase	Neyrinck et al. [80]
Sijunzi decoction	*Panax ginseng*, *Atractylodes macrocephala Koidz*, the sclerotium of the fungus, *Poria cocos*, *Glycyrrhiza uralensis fisch*	In vitro	↑*Lactobacillus*, *Pediococcus*, *Sutterella*; ↓*Paraprevotella*, *Bacteroides*, *Streptococcus*, *Clostridium*, *Ruminococcus*, *Butyricimonas*,	↑acetic acid, total acid ↓propionic acid, butyric acid	Immunomodulatory function	Gao et al. [81]
Resistant starch	Purple yam	High-fat diet	↑*Bifidobacteria*, *Lactobacillus*, *Coprococcus*, *Allobaculum*; ↓*Parabacteroides*, *Dorea*	None	Ameliorate lipid metabolism	Li et al. [82]
Ethanol extract of *Ganoderma lucidum* (GL95)	*G. lucidum*	High-fat diet	↑*Alistipes*, *Peptococcaceae*, *Defluviitalea* and *Alloprevotella*; ↓*Phascolarctobacterium*, *Clostridium XVIII*	↑Bile acid, SCFAs, HMGCR, CYP7A1, PPAR*α*, ApoB; ↓FAS, ACAT2, SREBP-1C, HMGCR	Improve lipid metabolism	Guo et al. [83]
Ursolic acid	Natural pentacyclic triterpenoid compound derived from Chinese medicine plants	CCl4-induced liver fibrosis	↑*Firmicutes*, *Bacteroidales*, and *Lachnospiraceae* ↓*Verrucomicrobia*	None	Inhibit the NOX4/ROS and RhoA/ROCK1 signalling pathways, reverses liver damage and fibrosis	Wan et al. [84]

SCFAs: short-chain fatty acids; NAFLD: nonalcoholic fatty liver disease; HFD: high-fat diet; LPS: lipopolysaccharide; MAPK: mitogen-activated protein kinase.

## Abbreviations

TCM: Traditional Chinese medicine
NAFLD: Nonalcoholic fatty liver disease
LPS: Lipopolysaccharide
CHB: Chronic hepatitis B
CHC: Chronic hepatitis C
HCV: Hepatitis C virus
HBV: Hepatitis B virus
HBVCLD: HBV-induced chronic liver disease
HCVCLD: HCV-induced chronic liver disease
SCFAs: Short-chain fatty acids
B/E: Bifidobacterium/Enterobacteriaceae ratio
HFD: High-fat diet
EnEth: Endogenous ethanol
BT: Bacteria translocation
ALF: Acute liver failure
SALF: Subacute liver failure
ACLF or SACLF: Slow-onset acute (subacute) liver failure
CLF: Chronic liver failure
HCC: Hepatocellular carcinoma
PAMPs: Pathogen-associated molecular patterns
DAMPs: Damage-related molecular patterns
SBP: Spontaneous bacterial peritonitis
HE: Hepatic encephalopathy
SBP: Spontaneous bacterial peritonitis
FFAs: Free fatty acids
HF: High-fructose diet.
